# Editorial: Rising stars in surgical oncology 2021

**DOI:** 10.3389/fonc.2023.1200078

**Published:** 2023-04-19

**Authors:** Massimiliano Veroux

**Affiliations:** ^1^ General Surgery and Organ Transplant Unit, Azienda Policlinico San Marco University of Catania, Catania, Italy; ^2^ Department of Medical and Surgical Sciences and Advanced Technologies, University of Catania, Catania, Italy

**Keywords:** surgical oncology, cancer, robotic nephrectomy, HIPEC (heated intraperitoneal chemotherapy), HCC, transcatheter arterial chemoembolization, hypersplenism, retroperitoneal sarcomas

Cancer continues to be a growing cause of mortality, with more than 23 million new cancer cases and 10 million cancer deaths globally in 2019, a 23% increase since 2010 (1, 2). Understanding the main risk factors contributing to the increase in incidence and mortality is the next challenge for oncologists and particularly for surgical oncologists, since only early identification and prompt treatment would decrease cancer mortality.

Probably no field has developed more in the last decade than surgical oncology, with the integration of new technologies, such as mini-invasive and robotic surgery, and multidisciplinary treatments, including interventional radiology.

This Research Topic, Rising Stars in Surgical Oncology 2021, offered the possibility to internationally recognized researchers in the early stages of their careers to present their latest results in the diagnosis and surgical treatment of cancer, together with the newer experimental findings in the field of surgical oncology.

Surgery is the mainstay treatment of most cancers and during the last decade many improvements in surgical oncology have been made, particularly with the development of mini-invasive techniques such as robotic surgery. In their manuscript, Li et al. evaluated the efficacy of three-dimensional computed tomography (CT) scans to identify the preoperative predictive factors for postoperative outcomes after robotic nephrectomy. They evaluated a new parameter, the tumor bed area, and found that it was significantly associated with a prolonged warm ischemia time, which is a well-known risk factor for the postoperative rise of serum creatinine and long-term renal insufficiency, and extent of surgery. This could aid better surgical planning for those patients with high complexities.

However, surgery may not be always completely curative and in patients with advanced disease, it must be combined with adjuvant therapies to reduce the recurrence and to improve survival. Yap et al. performed a meta-analysis to evaluate the effectiveness of hyperthermic intraperitoneal chemotherapy (HIPEC) in improving peritoneal recurrence rates in peritoneal metastases of different origins. This meta-analysis was not able to demonstrate a significant superiority of closed technique vs open technique for HIPEC, and highlighted the limited efficacy of HIPEC in ovarian and colon cancer, with only small effects on peritoneal metastases from gastric cancer.

In the setting of non-surgical treatment of advanced disease, interventional radiology has rapidly become an important tool in the management of many abdominal oncological diseases. In China, hepatocellular carcinoma (HCC) with cirrhosis from hepatitis B infection is common, and most patients present with liver cirrhosis and hypersplenism. Traditional treatment includes wedge resection of HCC lesions together with splenectomy. Partial splenic embolization (PSE) has recently emerged as an effective therapeutic modality for the treatment of hypersplenism secondary to chronic liver disease. Zhou et al. reported the first study investigating the role of transcatheter arterial chemoembolization (c-TACE) with drug-eluting microspheres with simultaneous PSE in the treatment of patients with HCC and hypersplenism. Ninety patients with HCC and hypersplenism were selected to receive c-TACE+ PSE (32 patients) or c-TACE only (58 patients). There was no significant difference in liver function and adverse events rate between the two groups, but c-TACE+PSE group displayed a significantly higher response rate and median overall survival.

Management of rare tumors represents a challenge due to the paucity of available data and to the limited therapeutical approaches. Therefore, the correct and early identification of prognostic factors may allow for a better definition of the treatment strategy for these tumors.

Lim et al. aimed to validate a new recurrence nomogram, named Sarculator, to predict the survival outcomes in an Asian population of patients with retroperitoneal sarcomas.

Retroperitoneal sarcomas are associated with a high risk of recurrence and cancer-related mortality, and the new nomogram Sarculator was able to predict the overall survival of patients with retroperitoneal sarcomas and would offer a new tool for a correct understanding of the biological behavior of sarcomas, thus allowing for risk stratification and a correct decision-making process.

Taken together, all these studies offer to readers a comprehensive view of the different aspects of the management of advanced oncological disease, with a special focus on the multidisciplinary approaches to diagnosis and surgical management that could allow, in principle, for timely diagnosis and treatment, thus allowing, hopefully, a better long-term outcome.

## Author contributions

MV: edited the Research Topic, substantial contributions to the conception or design of the topic; wrote the editorial.
